# Melatonin Alleviates High-Fructose-Induced Renal Injury in Male Mice, Which Might Be Associated with the Regulation of Mitophagy and Fatty Acid Oxidation

**DOI:** 10.3390/nu18010068

**Published:** 2025-12-25

**Authors:** Yanzhen Ma, Dan Sun, Yixian Bai, Weiheng Liu, Xue Bai, Zhikang Liu, Tian Kong, Peng Wang, Xi Liang, Zhe Zhang, Hui Liang, Huaqi Zhang

**Affiliations:** 1 Department of Nutrition and Food Hygiene, School of Public Health, Qingdao University, 308 Ningxia Road, Qingdao 266071, China; mayanzhen97@163.com (Y.M.); sundan202409@163.com (D.S.); bai_yixian@163.com (Y.B.); 18648646729@163.com (W.L.); snowxue216@126.com (X.B.); 19826531768@163.com (Z.L.); wpeng@qdu.edu.cn (P.W.); liangxi6029@163.com (X.L.); qdlianghui@126.com (H.L.); 2 Qingdao Shibei District Center for Disease Control and Prevention, No. 18 Liaoyang West Road, Shibei District, Qingdao 266033, China; kt0504@163.com; 3 College of Food Science and Engineering, Ocean University of China, Qingdao 266000, China; zhangzhe1613@ouc.edu.cn

**Keywords:** melatonin, high fructose, renal injury, mitophagy, fatty acid oxidation

## Abstract

**Objective**: To explore the preventive effect and mechanism of melatonin on high-fructose-induced renal injury in mice. **Methods**: A total of forty male C57BL/6J mice aged six weeks were randomly assigned to four groups: control group (CON), melatonin group (MLT), fructose group (FRU), and fructose + melatonin group (FRU + MLT). The concentration of the fructose solution was 30%, and the dose of melatonin was 10 mg/kg/day by intragastric administration. The experiment lasts for 10 weeks. **Results**: Liquid intake and energy intake were comparable between the FRU and FRU + MLT, both of which were significantly higher than that in the CON and MLT. MLT inhibited fructose-induced increased levels in serum creatinine (Cre), serum urea nitrogen (BUN), serum uric acid (UA), serum triglyceride (TG), renal kidney injury molecule-1 (KIM-1), and renal TG. Hematoxylin and Eosin (H&E) staining and Oil Red O (ORO) staining showed that MLT alleviated renal tubular dilatation, loss of brush border, epithelial cell detachment and lipid accumulation. Transmission electron microscope (TEM) observations showed that MLT increased autophagic vacuoles among mitochondria. Western blot analysis showed that, compared with the FRU, the FRU + MLT had elevated expression of AMP-activated protein kinase (AMPK) phosphorylation, along with a significant increase in the expression of its downstream mitophagy-related proteins (including PINK1, Parkin, LC3 II, and Beclin1), whereas the expression of p62 was markedly decreased. Furthermore, the expression levels of FAO-related proteins (including PPARα and CPT1A) in the FRU + MLT were significantly upregulated. **Conclusions**: MLT alleviates renal injury caused by high-fructose exposure in male mice and its mechanism might be associated with the regulation of mitophagy and fatty acid oxidation.

## 1. Introduction

Fructose, a monosaccharide naturally present in fruits, serves as the primary component of high-fructose corn syrup and is frequently employed as a sweetener in beverages, baked products, and confectionery [1,2,3]. In recent decades, the intake of fructose has increased dramatically, especially in Western countries [4,5]. High fructose exposure has been proven to cause renal injury [6,7]. Animal studies suggest that feeding mice a 10–30% fructose solution for about 10 consecutive weeks can lead to tubular dilation, renal lipid accumulation, and renal dysfunction [8,9,10].

The kidney has abundant mitochondria, and the normal activity of these organelles is crucial for a well-functioning kidney [11,12]. Mitochondrial dysfunction is implicated in the pathogenesis of renal injury induced by high-fructose diets [13,14]. Generally, when renal cells are in a state of stress or exposed to adverse stimuli, the dynamin-related protein 1 (Drp1) in the kidneys is abnormally upregulated, which accelerates mitochondrial fission. In contrast, the reduced expression of mitofusin1/2 (Mfn1/Mfn2) and optic atrophy 1 (OPA1) impairs mitochondrial fusion [15,16,17]. This imbalance ultimately results in excessive mitochondrial fragmentation and structural disorganization [18,19]. Damaged mitochondria recruit and accumulate PTEN-induced kinase 1 (PINK1). PINK1 further recruits Parkin to ubiquitinate mitochondrial proteins, and this process marks damaged mitochondria for autophagic clearance to preserve mitochondrial homeostasis [20]. Mitophagy is widely recognized as a protective mechanism against kidney dysfunction [20,21,22]. Studies have demonstrated that high-fructose exposure not only triggers excessive mitochondrial fragmentation and structural disorder but also inhibits mitophagy [13]. Inhibition of mitophagy results in the failure to clear damaged mitochondria, which aggravates mitochondrial dysfunction and subsequently contributes to renal injury [23,24]. Additionally, high-fructose exposure can significantly impair the fatty acid oxidation (FAO) function in renal proximal tubular epithelial cells, leading to lipid accumulation in the kidneys [25,26,27]. As a key mitochondrial-dependent energy metabolism pathway, the impairment of FAO manifests as mitochondrial dysfunction: it not only reduces mitochondrial ATP production, failing to meet the physiological needs of renal cells, but also promotes the massive generation of reactive oxygen species (ROS) and accelerates the progression of renal injury [28,29]. Therefore, activating mitophagy and regulating mitochondrial FAO may represent effective strategies to alleviate high-fructose-induced renal injury.

Melatonin (MLT) is an indoleamine primarily synthesized in the pineal gland, with trace amounts also found in cherries and walnuts [30,31]. Nowadays, MLT has been used as a dietary supplement for the prevention and adjuvant treatment of conditions such as diabetes, cardiovascular disease, and chronic neurodegenerative disorders [32,33,34]. Recently, the impact of MLT on kidney diseases has attracted attention. Population studies found that patients with chronic kidney disease were accompanied by a decrease in endogenous MLT levels [35]. Animal experiments indicated that MLT intervention could alleviate the senescence and apoptosis of renal cortical proximal epithelial tubule (HK-2) cells in patients with diabetic nephropathy [36]. MLT plays a preventive and therapeutic role in various diseases by regulating the expression of AMPK [37,38]. AMPK has been confirmed to directly participate in the regulation of mitophagy [39]. Additionally, AMPK is also involved in modulating FAO processes [40]. Therefore, it is reasonable to speculate that MLT may ameliorate kidney damage caused by high fructose exposure through AMPK-mediated mitophagy and FAO.

In this study, C57BL/6J mice were allowed free access to a 30% fructose solution and 10 mg/kg MLT was administered via daily intragastric delivery throughout a 10-week experimental period to investigate the preventive efficacy of MLT against high-fructose-induced renal injury. It is the first time to explore its underlying mechanism through the pathways of mitophagy and FAO.

## 2. Materials and Methods

### 2.1. Animals and Experimental Design

This study was approved by the Experimental Animal Welfare and Ethics Committee of Qingdao University (Approval No. 20231102C574020240113054; Approval Date: 23 September 2024). A power analysis (α = 0.05, power = 0.8) based on the pilot data indicated that a sample size of *n* = 9 per group would be sufficient to detect a significant intervention effect. A group size of *n* = 10 was used to accommodate any unexpected losses. A total of forty male C57BL/6 mice, aged six weeks and weighing 20 ± 2 g, were purchased from Beijing Huafukang Biotechnology Co, and housed at 22–24 °C with 40–60% humidity under a normal circadian rhythm.

**Figure 1:**
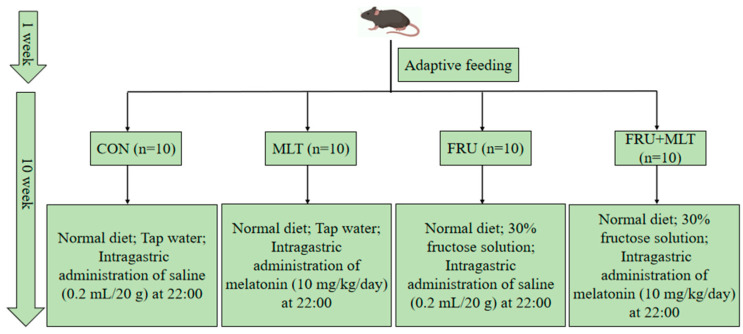
Experimental grouping and interventions of the study.

After the acclimation for 1 week, all mice were randomly assigned to four groups after matching for body weight with 10 mice in each group. All subsequent procedures and analyses were performed by investigators blinded to the group allocation. All four groups of animals were fed the same standard diet, and the detailed dietary formulations are provided in Table S1. In the control group (CON), the mice were given tap water and treated intragastrically with normal saline (0.2 mL/20 g). In the melatonin group (MLT), the mice were given tap water and treated intragastrically with 10 mg/kg/day MLT. In the fructose group (FRU), the mice were given 30% (*w*/*v*) fructose solution and treated intragastrically with normal saline (0.2 mL/20 g). In the fructose + melatonin group (FRU + MLT), the mice were given 30% (*w*/*v*) fructose solution and treated intragastrically with 10 mg/kg/day MLT. Intragastric administration was conducted at 22:00 daily, consistent with previously published studies [41]. Animals in each group had free access to tap water or a 30% fructose solution, respectively. MLT (CAS Number: 73-31-4) was purchased from Sigma-Aldrich Chemical Co. (St. Louis, MO, USA) and dissolved in 0.9% normal saline before administration. Fructose (purity ≥ 99%) was obtained from Macklin Biochemical Technology Co., Ltd. (Shanghai, China). The experiment lasted 10 weeks, with daily measurements of food and liquid intake. The specific intervention protocol is illustrated in Figure 1.

After 10 weeks, all animals were fasted for 12 h and were anesthetized for blood collection and then euthanized. Kidneys were rapidly excised and weighed. Tissues were processed as follows: the right kidneys were used for hematoxylin and eosin (H&E) staining, Oil Red O (ORO) staining, BODIPY staining and immunofluorescence. The left kidneys were partially used for ROS detection, another portion was for transmission electron microscopy (TEM), and the remaining part was stored at −80 °C until use.

### 2.2. Serum Biochemical Analysis

Serum levels of triglyceride (TG), total cholesterol (TC), creatinine (Cre), urea nitrogen (BUN), and uric acid (UA) were measured by using an AU5400 automatic biochemical analyzer (Beckman, Los Angeles, CA, USA).

### 2.3. Biochemical and Molecular Analyses of Renal Tissues

Kidney tissues were homogenized in normal saline at a ratio of 1:9 (g:mL) and then centrifuged at 2500 rpm for 15 min. After centrifugation, the supernatant was collected to determine the concentrations of kidney injury molecule-1 (KIM-1, a renal injury marker; Cat# H436-1-1), TG (Cat# A110-1-1), IL-1β (Cat# H002-1-1), IL-6 (Cat# H007-1-1), TNFα (Cat# H052-1-1), and MDA (Cat#A003-1-1), as well as the enzymatic activities of SOD (Cat# A001-1-1), GSH-Px (Cat# A005-1-1) and CAT (Cat# A007-1-1) in kidney tissues, which were measured with corresponding ELISA kits (Nanjing Jiancheng Biological Engineering Research Institute, Nanjing, China). All samples were assayed in triplicate.

The Western blot scheme follows the standard procedure used in a previous study [42]. Protein extracts were prepared from three independent biological samples per group. Primary antibodies used in this experiment targeted three categories of proteins: (1) mitophagy-related proteins, including AMPK (Cat# ab32047), p-AMPK (Cat# ab133448), PINK1 (Cat# ab216144), Parkin (Cat# ab77924), LC3 II (Cat# ab192890), Beclin1 (Cat# ab207612), and P62 (Cat# ab109012); (2) mitochondrial dynamics-related proteins, including FIS1 (Cat# ab156865), Drp1 (Cat# ab184247), Mfn1 (Cat# ab221661), Mfn2 (Cat# ab124773) and OPA1 (Cat# ab157457); and (3) FAO-related proteins, including PPARα (Cat# ab126285) and CPT1A (Cat# ab128568). β-Actin (Cat# ab6276) was used as the internal loading control. All antibodies were purchased from Abcam plc, Cambridge, UK.

### 2.4. Renal Ultrastructure Observation

Left kidney tissues were fixed overnight, followed by dehydration, embedding, polymerization, sectioning (50 nm), and staining. Samples were visualized via JEM-1200EX Transmission Electron Microscope (TEM; JEOL, Tokyo, Japan).

### 2.5. H&E Staining

Right kidney tissues were first immersed in 4% paraformaldehyde solution for 24 consecutive hours; after fixation, they were embedded in paraffin and further sectioned into 5-μm-thick slices. Once sectioned, the renal slices were stained using H&E. Renal histopathological alterations were then visualized and imaged under a light microscope (Olympus Corporation, Tokyo, Japan). Three non-consecutive kidney sections per mouse were analyzed.

### 2.6. ORO Staining

The cryosections of fresh right kidney tissue were stained with the ORO solution and washed using 60% isopropanol and distilled water. Subsequently, nuclei were counterstained with hematoxylin and observed under a BX60 light microscope (Olympus, Tokyo, Japan). For each mouse, three non-consecutive sections were analyzed to ensure representative sampling.

### 2.7. BODIPY Staining

Fresh right renal cryosections were first immersed in 4% paraformaldehyde solution for 10 min for fixation, then rinsed three times with PBS buffer. Subsequently, the sections were incubated at room temperature for 60 min with 5 μg/mL of BODIPY 493/503 supplied by Life Technologies (Carlsbad, CA, USA). Following additional rinsing, the sections were counterstained using DAPI and then mounted onto slides. Staining results were visualized using an Olympus confocal microscope (Olympus Corporation, Tokyo, Japan).

### 2.8. Immunofluorescence

Renal paraffin sections (4 μm thick) were employed for immunofluorescence staining. First, the sections were permeabilized using 0.3% Triton X-100 and then blocked with 4% bovine serum albumin (BSA). After blocking, the sections were incubated with primary antibodies at 4 °C overnight, followed by incubation with secondary antibodies for 50 min. The primary antibodies utilized included TOM20 (1:1000 dilution, Servicebio (Wuhan, China)) and LC3 (1:500 dilution, Servicebio); additionally, DAPI (Servicebio) was used for nuclear counterstaining. Regions with fluorescent signals were visualized and imaged using a fluorescence microscope (Olympus Corporation, Tokyo, Japan). Ten pictures were taken at randomly chosen fields from each section.

### 2.9. Renal ROS Assay

Kidney tissue homogenates were diluted with PBS solution and then mixed with 2,7-dichlorofluorescin diacetate (Elabscience, Wuhan, China) to incubate for 30 min. After rinsing with PBS and centrifugation, the supernatant was discarded to collect renal cells, resuspended in 0.5 mL of PBS solution, and then the fluorescence value was measured by flow cytometry.

### 2.10. Renal ATP Measurement and Mitochondrial DNA Copy Number Detection

Renal ATP concentrations were determined via a commercial ATP assay kit (Cat# A095-2-1, Jiancheng Bioengineering Institute, Nanjing, China) per the kit’s instructions. Mitochondrial DNA (mtDNA) copy number was quantified via real-time polymerase chain reaction (RT-PCR) using a SYBR Green kit (Cat# RR82LR, Takara Bio Inc., Tokyo, Japan). All qPCR reactions were run in triplicate for each independent sample.

### 2.11. Statistical Analysis

SPSS 23.0 (SPSS, Chicago, IL, USA) was used for statistical analysis. Data are presented as means ± standard deviation (SD) if normally distributed, or as median with interquartile range (IQR) otherwise. Normality (Shapiro–Wilk test) and homogeneity of variances (Levene’s test) were assessed. For multi-group comparisons meeting both assumptions, one-way ANOVA with Tukey’s post hoc test was used. If the equal variance assumption was violated, Welch’s ANOVA with the Games-Howell test was applied. Non-normally distributed data were analyzed by the Kruskal–Wallis test followed by Dunn’s test. *p* < 0.05 was considered statistically significant.

## 3. Results

### 3.1. Effect of MLT on Body Weight and Kidney Index

**Figure 2:**
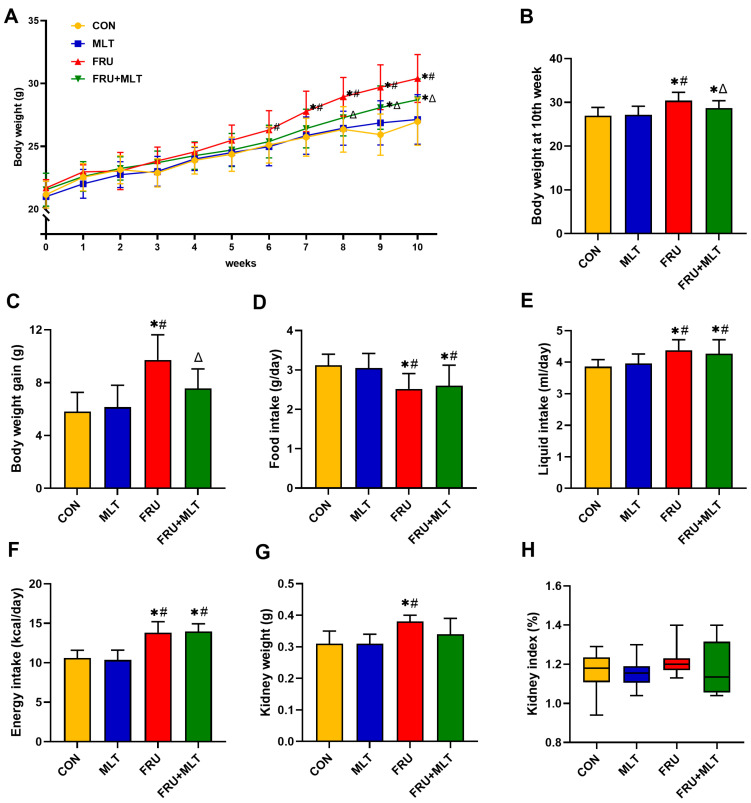
Effect of MLT on body weight and kidney index. (**A**) Body weight. (**B**) Body weight at 10th week. (**C**) Body weight gain. (**D**) Food intake. (**E**) Liquid intake. (**F**) Energy intake. (**G**) Kidney weight. (**H**) Kidney index. In panels (**A**–**G**), data are presented as mean ± SD (*n* = 10). In panel (**H**), data are presented as median with IQR (*n* = 10). * *p* < 0.05 vs. CON, # *p* < 0.05 vs. MLT, Δ *p* < 0.05 vs. FRU.

Figure 2A illustrated that no statistically significant differences were observed in initial body weights across the four experimental groups (*p* > 0.05). Compared to CON, FRU showed a significant elevation in both body weight at the 10th week and total body weight gain; in contrast, FRU + MLT exhibited a marked reduction in these two indices relative to FRU (*p* < 0.05; Figure 2B,C). Additionally, food consumption in FRU and FRU + MLT was notably lower compared to CON and MLT (*p* < 0.05; Figure 2D). In contrast, FRU and FRU + MLT had significantly higher liquid intake and energy intake than CON and MLT (*p* < 0.05; Figure 2E,F). As shown in Figure 2G, compared with the CON, the kidney weight in the FRU was significantly increased (*p* < 0.05). There were no significant differences in kidney index among the four groups (*p* > 0.05; Figure 2H).

### 3.2. Effect of MLT on Serum Biochemical Indicators

**Figure 3:**
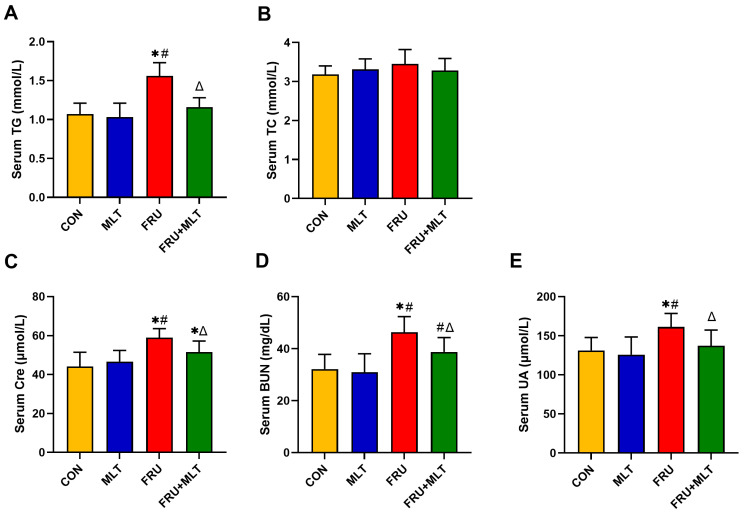
Effect of MLT on serum biochemical indicators. (**A**) Serum TG. (**B**) Serum TC. (**C**) Serum Cre. (**D**) Serum BUN. (**E**) Serum UA. The data are presented as mean ± SD (*n* = 10). * *p* < 0.05 vs. CON, # *p* < 0.05 vs. MLT, Δ *p* < 0.05 vs. FRU.

In Figure 3A, the serum level of TG was significantly higher in FRU than in CON (*p* < 0.05). In comparison to FRU, FRU + MLT had a markedly lowered serum level of TG (*p* < 0.05). No significant differences were observed in serum TC levels among the four groups (*p* > 0.05; Figure 3B). The serum Cre, BUN and UA levels in FRU were significantly higher than those in CON (*p* < 0.05). MLT supplementation significantly reduced the increase in Cre, BUN and UA when compared to FRU (*p* < 0.05; Figure 3C–E).

### 3.3. Effect of MLT on Renal Injury and Renal Lipid Accumulation

**Figure 4:**
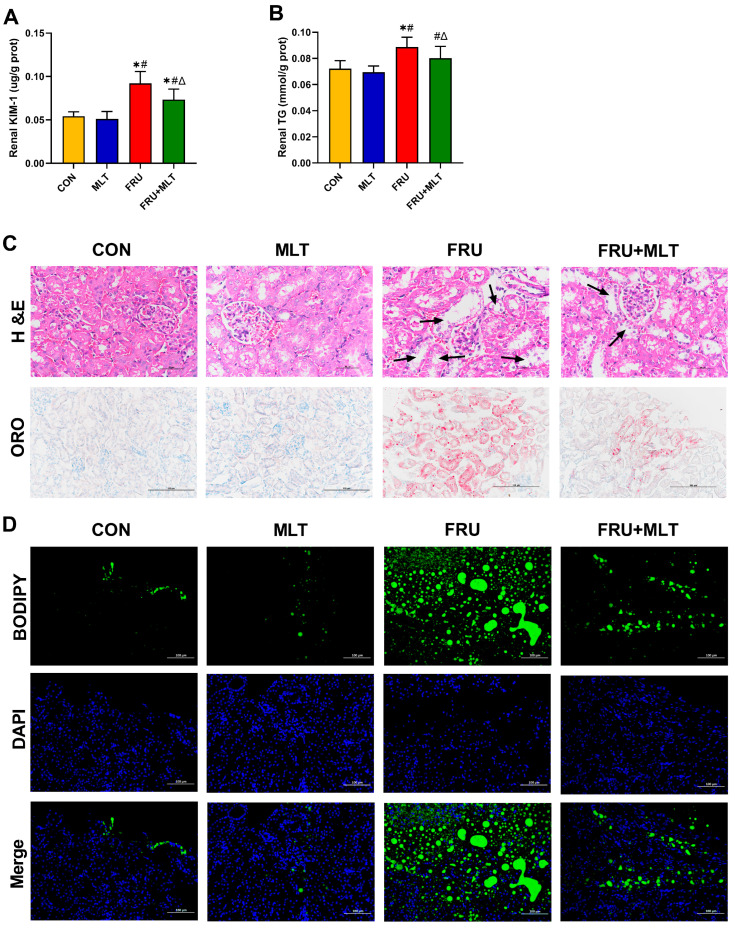
Effect of MLT on renal injury and renal lipid accumulation. (**A**) Renal KIM-1. (**B**) Renal TG. (**C**) H&E and ORO staining of Kidney (40×, 20×, scale bars: 50 µm, 100 µm) (black arrowhead: renal tubular dilation). (**D**) BODIPY Staining (20×, scale bars: 100 µm) Lipids stained green and nuclei stained blue. Data are presented as mean ± SD (*n* = 10). * *p* < 0.05 vs. CON, # *p* < 0.05 vs. MLT, Δ *p* < 0.05 vs. FRU.

KIM-1 is a biomarker of renal injury. Compared with CON, FRU showed an elevated renal KIM-1 level; in contrast, FRU + MLT displayed a marked reduction in renal KIM-1 level relative to FRU (*p* < 0.05; Figure 4A). Relative to CON, FRU had a significantly higher renal TG level, while FRU + MLT exhibited a significantly normalized renal TG level compared to FRU (*p* < 0.05; Figure 4B). H&E, ORO and BODIPY staining assays were employed to further assess renal pathological alterations and lipid accumulation. In Figure 3C, H&E staining showed normal morphology in CON. Renal tubular dilatation, loss of brush border and epithelial cell detachment were observed in FRU, while the above pathological changes in FRU + MLT were significantly improved. ORO and BODIPY staining showed obvious lipid accumulation in the kidneys of FRU, while the lipid accumulation in FRU + MLT was significantly improved (Figure 4C,D).

### 3.4. Effect of MLT on Renal ROS Accumulation, Renal Oxidative Stress and Renal Inflammation

**Figure 5:**
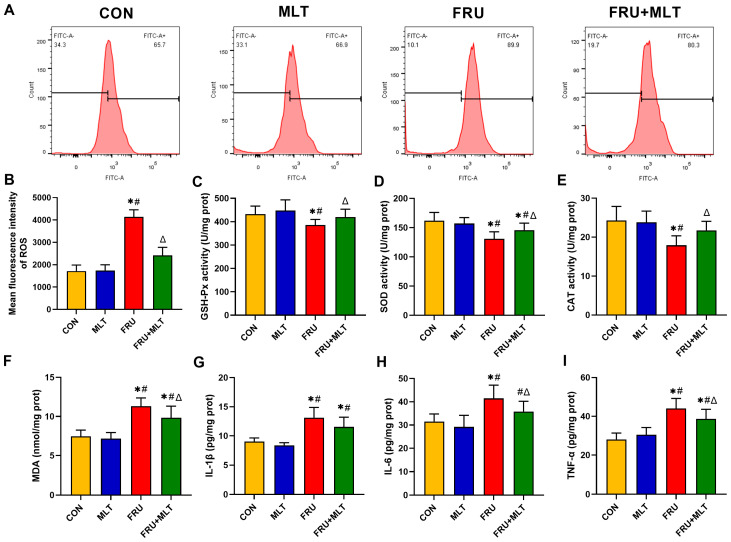
Effect of MLT on renal ROS accumulation, renal oxidative stress and renal inflammation. (**A**) Flow cytometry map of ROS. (**B**) Mean fluorescence intensity of ROS. (**C**) GSH-Px activity. (**D**) SOD activity. (**E**) CAT activity. (**F**) MDA content. (**G**) IL-1β content. (**H**) IL-6 content. (**I**) TNF-α content. Data are presented as mean ± SD (*n* = 6 per group for ROS flow cytometry and mean fluorescence intensity; *n* = 10 per group for MDA, GSH-Px, SOD, CAT, IL-1β, IL-6, and TNF-α). * *p* < 0.05 vs. CON, # *p* < 0.05 vs. MLT, Δ *p* < 0.05 vs. FRU.

Compared with CON, FRU exhibited a significant elevation in the mean fluorescence intensity of ROS (*p* < 0.05). Relative to FRU, FRU + MLT showed a marked reduction in ROS mean fluorescence intensity (*p* < 0.05; Figure 5B). Additionally, when compared to CON, the FRU had an observable decrease in renal GSH-Px, SOD, and CAT activity, alongside a significant increase in renal MDA content (*p* < 0.05). Furthermore, the FRU + MLT displayed a significant decrease in renal MDA content and a noticeable increase in renal GSH-Px, SOD, and CAT levels relative to the FRU (*p* < 0.05; Figure 5C–F). As shown in Figure 5G–I, the levels of renal IL-1β, IL-6, and TNF-α in the FRU significantly elevated compared with the CON (*p* < 0.05), while MLT treatment significantly reversed the elevations of renal IL-6 and TNF-α in FRU (*p* < 0.05).

### 3.5. Effect of MLT on Renal Mitochondrial Damage and Renal Mitophagy

**Figure 6:**
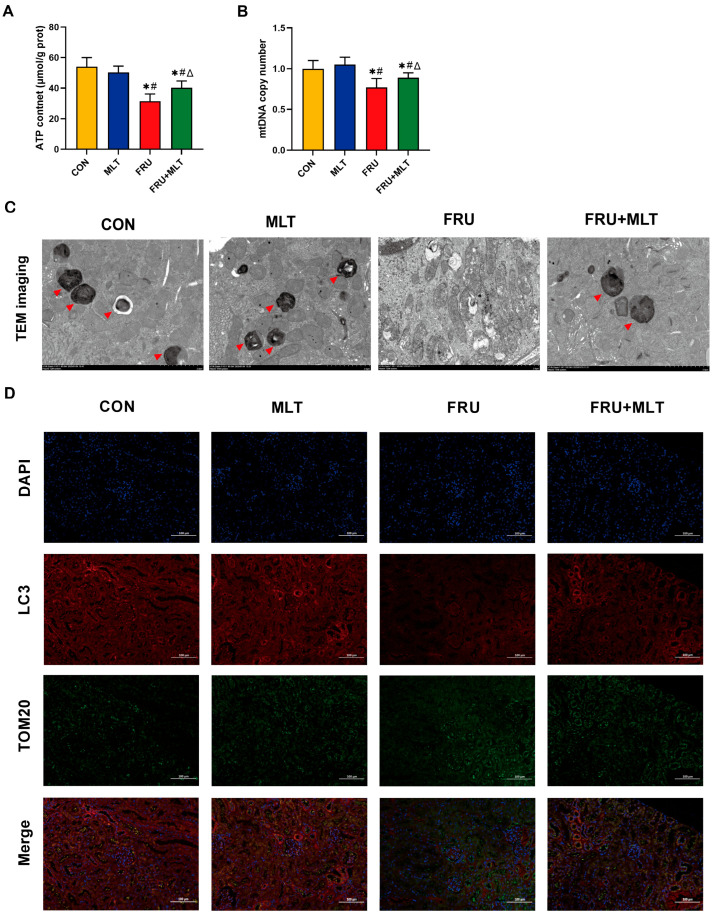
Effect of MLT on renal mitochondrial damage and renal mitophagy. (**A**) ATP content. (**B**) Mitochondrial DNA copy number. (**C**) TEM images in the kidney tissues (Red arrowhead indicates mitophagosomes. Scale bar: 2 μm). (**D**) Representative immunofluorescent images of fluorescence intensity for LC3 (Red, mitophagosome marker) and TOM20 (Green, mitochondrial outer membrane marker). Nuclei are counterstained with DAPI (Blue). Scale bar: 100 μm. Data are presented as mean ± SD (*n* = 10 per group for ATP and mitochondrial DNA copy number; *n* = 6 per group for TEM and immunofluorescent images). * *p* < 0.05 vs. CON, # *p* < 0.05 vs. MLT, Δ *p* < 0.05 vs. FRU.

Figure 6A,B demonstrate that FRU had significantly lower ATP content and mtDNA copy number than CON (*p* < 0.05). Relative to FRU, FRU + MLT exhibited a significant increase in both mtDNA copy number and ATP quantity (*p* < 0.05). TEM observations revealed that the mitochondrial cristae in the renal tissues of CON had clear and intact structures. However, mice in FRU exhibited significant mitochondrial damage, manifesting as mitochondrial swelling, almost complete loss of cristae structures, and a decrease in observable mitophagosomes. In contrast, renal mitophagy impairment was reversed in FRU + MLT (Figure 6C). Moreover, the colocalization between LC3 and TOM20 was notably reduced in FRU compared to CON. When compared to FRU, the colocalization of LC3 and TOM20 was significantly enhanced in FRU + MLT. MLT promoted the colocalization of LC3 and mitochondrial marker TOM20 as shown by the increase in merged intensity of LC3 with TOM20 puncta (Figure 6D).

### 3.6. Effect of MLT on Renal Mitophagy, Mitochondrial Dynamics and FAO-Related Protein Expression

**Figure 7:**
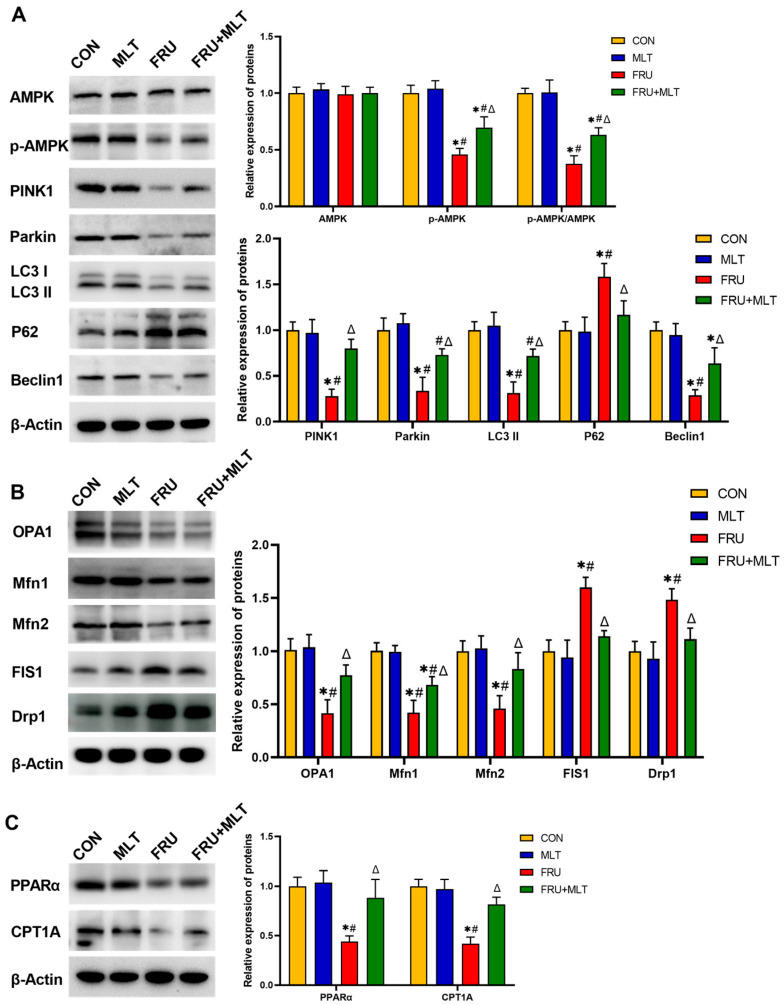
Effect of MLT on renal mitophagy, mitochondrial dynamics and FAO-related protein expression. (**A**) Protein expression levels related to renal mitophagy. (**B**) Protein expression levels related to renal mitochondrial dynamics. (**C**) Protein expression levels related to renal FAO. The data are presented as mean ± SD (*n* = 3). * *p* < 0.05 vs. CON, # *p* < 0.05 vs. MLT, Δ *p* < 0.05 vs. FRU.

Figure 7A illustrated that, compared to CON, FRU exhibited significant reductions in the protein expression levels of p-AMPK (*p* < 0.001, Hedges’ g = −4.02), PINK1 (*p* < 0.001, Hedges’ g = −5.21), Parkin (*p* < 0.001, Hedges’ g = −3.19), LC3 II (*p* < 0.001, Hedges’ g = −4.17), and Beclin1 (*p* = 0.023, Hedges’ g = −2.83), accompanied by a marked increase in P62 expression (*p* = 0.004, Hedges’ g = 5.72). Relative to FRU, FRU + MLT showed significantly higher protein expression levels of p-AMPK (*p* = 0.017, Hedges’ g = 1.98), PINK1 (*p* = 0.001, Hedges’ g = 4.15), Parkin (*p* = 0.014, Hedges’ g = 2.71), LC3 II (*p* < 0.001, Hedges’ g = 3.22), and Beclin1 (*p* = 0.023, Hedges’ g = 1.97), together with a decrease in P62 (*p* = 0.023, Hedges’ g = −2.18). Figure 7B demonstrated that when compared to CON, FRU showed notably decreased OPA1 (*p* < 0.001, Hedges’ g = −3.47), Mfn1 (*p* < 0.001, Hedges’ g = −4.23), and Mfn2 (*p* = 0.003, Hedges’ g = −3.68) expression and significantly increased FIS1 (*p* < 0.001, Hedges’ g = 3.27) and Drp1 (*p* = 0.004, Hedges’ g = 2.98) expression. Relative to FRU, FRU + MLT displayed a significant increase in OPA1 (*p* = 0.019, Hedges’ g = 2.51), Mfn1 (*p* = 0.022, Hedges’ g = 1.89) and Mfn2 (*p* = 0.025, Hedges’ g = 2.14) expression and a notable reduction in the expression levels of FIS1 (*p* = 0.004, Hedges’ g = −3.82) and Drp1 (*p* = 0.019, Hedges’ g = −2.87). In terms of PPARα and CPT1A expression (Figure 6C), relative to CON, FRU exhibited dramatically lower levels of PPARα (*p* = 0.002, Hedges’ g = −4.45) and CPT1A (*p* < 0.001, Hedges’ g = −0.92). When contrasted with FRU, FRU + MLT showed markedly higher PPARα (*p* = 0.009, Hedges’ g = 2.56) and CPT1A (*p* = 0.001, Hedges’ g = 0.53) expression.

## 4. Discussion

Fructose is commonly used as a sweetener and is widely applied in processed foods. Over the years, with the increased consumption of processed foods, the health risks caused by high-fructose exposure have attracted increasing attention [43,44,45,46]. Long-term high-fructose exposure has been proven to induce renal injury. For instance, animal studies have found that drinking a 30% fructose solution for about 10 consecutive weeks induces hyperuricemic nephropathy in mice [8,9,10]. Proactively preventing and managing renal injury caused by high-fructose exposure is of great significance. MLT is an indoleamine primarily synthesized in the pineal gland, and it is present in foods such as cherries and walnuts [47,48]. Nowadays, due to its well-documented anti-inflammatory, antioxidant, and immune-regulatory effects, MLT has been developed as a therapeutic agent or dietary supplement and used in the treatment of sleep disorders, metabolic syndrome, etc. [49,50,51]. MLT has also been explored for the treatment of various kidney diseases [52,53]. Previous research has shown that MLT exerts an ameliorative effect on diabetic nephropathy, as it can reduce renal cell apoptosis by regulating STAT3 phosphorylation [36]. Moreover, MLT can alleviate diclofenac-induced acute kidney injury through promoting the signaling of Nrf2/HO-1 [54]. MLT also exhibits excellent preventive and therapeutic effects on health issues induced by high-fructose exposure, such as metabolic syndrome, hepatic steatosis [55,56]. However, research on the preventive effect of MLT against high fructose-induced renal injury remains rather limited to date.

To elucidate the preventive effect of MLT on high-fructose-induced renal injury, we divided mice into four groups: CON, MLT, FRU and FRU + MLT. The concentration of the fructose solution is 30%, which was consistent with the intervention doses reported in previous studies [9,10]. The administration of 10 mg/kg of MLT via gavage was reported to effectively alleviate non-alcoholic fatty liver disease induced by a high-fat and high-fructose diet in mice [57], and it can ameliorate anxiety and depression-like behaviors and modulate proteomic changes in triple transgenic mice of Alzheimer’s disease [58]. Moreover, 10 mg/kg MLT attenuated retinal neovascularization and neuroglial dysfunction by inhibiting the HIF-1α-VEGF pathway in oxygen-induced retinopathy mice [59]. Additionally, 10 mg/kg of MLT also exerts protective effects against cadmium exposure-induced and ciprofloxacin-induced nephrotoxicity in previous animal studies [60,61]. Therefore, in this study, we also selected an MLT dose of 10 mg/kg. After 10 weeks of intervention, the serum Cre, BUN and UA levels in the FRU were significantly increased by 33.44%, 44.00%, and 22.98%, respectively, compared with those in the CON. Meanwhile, the renal KIM-1 level was significantly elevated by 69.8% relative to the CON. These findings suggested that mice in the FRU developed mild renal dysfunction. The serum Cre, BUN, UA levels and renal KIM-1 level in the FRU + MLT were significantly decreased compared with those in the FRU. Pathological examination of the kidneys can provide more direct evidence for the assessment of renal injury. In FRU, renal tubular dilatation, loss of brush border, and epithelial cell detachment were observed, while these pathological changes were significantly alleviated in FRU + MLT. The histopathological changes observed were consistent with the alterations in serum renal function indicators and renal KIM-1 levels. Based on the above results, we found that high-fructose exposure induces renal injury, while MLT effectively mitigates it. The mechanism by which MLT mitigates high fructose-induced renal injury has aroused our considerable interest.

The kidney is an organ with high energy demands and is highly dependent on mitochondria for energy production. Mitophagy, a selective autophagic process, eliminates damaged or dysfunctional mitochondria to sustain cellular survival and growth while preventing further cellular damage. In this process, the accumulation of PINK1 signals the recruitment of Parkin to the mitochondrial surface, where Parkin ubiquitinates the damaged mitochondria. P62 then binds to polyubiquitinated mitochondrial proteins and recruits LC3, a marker of autophagosomes. TEM observations revealed dysfunctional autolysosomes in renal tissues of FRU, indicating the presence of mitophagy impairment in the kidneys. Notably, mitochondrial ATP production capacity, mtDNA copy number, and ROS levels serve as direct indicators of renal mitochondrial energy supply, biosynthetic stability, and oxidative stress status, respectively [62,63,64]. In FRU, mitochondrial ATP production and mtDNA copy number were decreased, while ROS levels were elevated. These results demonstrate that high-fructose exposure induces renal injury by disrupting mitochondrial dynamics and inhibiting mitophagy—a finding that aligns with the conclusions of prior research [9,65].

AMPK has been well established as a critical regulator of mitophagy [66]. For example, a recent study found decreased mitochondrial translocation and expression of PINK1 following AMPK inhibition in the renal tubular cell [67]. This finding suggests that AMPK phosphorylation serves as an upstream target of PINK1. In a series of in vivo and in vitro studies, MLT has been well-confirmed to promote AMPK phosphorylation. For example, MLT improves glucose homeostasis and insulin sensitivity by mitigating inflammation and activating AMPK signaling in a mouse model of sleep fragmentation67 [68]. Moreover, MLT can also inhibit oxalate-induced endoplasmic reticulum stress and apoptosis in HK-2 cells by activating the AMPK pathway68 [69]. MLT can exert antioxidant and anti-inflammatory effects by activating AMPK signaling [37,69]. In this study, compared with FRU, the expression level of AMPK phosphorylation in FRU + MLT was increased, and the expression levels of PINK1 and Parkin were also elevated accordingly. Compared with FRU, FRU + MLT had higher mitochondrial ATP production capability, increased mtDNA copy number, and decreased ROS level. Furthermore, the expressions of LC3, Beclin1, OPA1, Mfn1 and Mfn2 in FRU + MLT were upregulated, while P62, FIS1 and Drp1 were downregulated. The above results indicated that the alleviation effect of MLT on high fructose-induced renal injury might be associated with the regulation of mitochondrial homeostasis and mitophagy.

In the kidney, the normal functioning of glomerular filtration and tubular reabsorption and secretion relies on continuous ATP supply. FAO is the main ATP-producing pathway in renal cells [29]. High fructose exposure can impair FAO in the kidney by downregulating PPARα expression [70]. PPARα is a core transcriptional factor that regulates FAO, which can directly bind to the promoter regions of FAO-related genes (including CPT1A) and promote their expression. CPT1A is the rate-limiting enzyme of mitochondrial FAO [71]. In the case of high-fructose exposure, PPARα downregulates, and the expression of CPT1A decreases accordingly. Unoxidized fatty acids then accumulate in renal cells, leading to lipid accumulation in the kidney [72]. The lipotoxicity caused by massive lipid accumulation further triggers renal cell inflammation, oxidative stress and apoptosis, thereby promoting the renal injury [10]. Therefore, FAO inhibition is an important mechanism underlying high fructose-induced renal injury. In this study, ORO staining demonstrated severe lipid accumulation in the kidneys of FRU. The results of the renal TG level test in FRU corresponded with the ORO staining findings. Compared with CON, the PPARα and CPT1A expressions were reduced in FRU, which can be used to explain the phenomenon observed by ORO staining. In FRU + MLT, the PPARα and CPT1A expressions were marked increased and renal lipid accumulation was significantly improved. The FAO process mediated by PPARα is directly regulated by AMPK in the kidney [73]. Therefore, MLT might exert a regulatory effect on FAO by regulating AMPK, thereby alleviating renal lipid accumulation and ultimately improving renal injury.

There were some limitations in this study. Firstly, this study was conducted solely in male mice and without a positive control group. Although the exclusive use of male animals was made to minimize physiological variability associated with the estrous cycle in females, it precludes any assessment of sex-dependent effects. The lack of a positive control group limits our ability to benchmark the efficacy of our intervention against established therapies. While the findings demonstrated the protective effect of MLT on high-fructose-induced renal injury in male mice, their applicability to females and their relative therapeutic potency require validation in more comprehensive future studies. Secondly, a high-concentration fructose solution (30%) was selected in this study to ensure robust and reproducible induction of renal injury within a controlled period. Although this approach is well-established and instrumental for mechanistic investigation, it is much higher than the fructose consumption level in human daily life. Future translational studies using fructose levels related to human consumption can bridge this gap. Thirdly, the absence of creatinine clearance, albuminuria, and highly specific protein markers for renal tubular injury limits the ability of this study to precisely quantify functional decline and delineate the primary site of renal injury. Nevertheless, the concordance of the static serum biomarkers (Cre, BUN, UA) with histopathological alterations observed in H&E staining, ORO staining, and TEM provides compelling evidence for renal injury and the protective effect of MLT. Fourthly, this study has a mechanistic limitation. In the FRU + MLT of this study, the observed improvement of mitophagy and FAO was highly consistent with the activation of AMPK, which aligns with the well-established role of AMPK as a master regulator of these processes. Given the extensive in vitro studies confirming MLT as a direct AMPK activator, it is a highly plausible mediator. However, a sequential causality from MLT to AMPK activation to downstream effects remains to be fully established. Moreover, the evidence supporting renal mitophagy and FAO mainly relies on a limited set of molecular markers assessed by Western blot (with a sample size of *n* = 3 per group) rather than functional assays. Future studies with larger sample sizes are warranted to increase statistical power and validate these molecular findings. In addition, employing AMPK-knockout models or specific inhibitors to confirm AMPK as the key upstream mediator. Subsequently, direct functional assays—such as measurements of mitophagic flux and mitochondrial FAO rates—will be essential to establish a definitive causal link between AMPK activation, downstream molecular adaptations, and the observed renoprotective phenotype.

The antioxidant, anti-inflammatory, immune regulatory, and potential anti-tumor effects of MLT make it of great value in disease prevention and adjuvant therapy. Nowadays, MLT has been applied in various human disease models and preliminary evidence has been obtained. For instance, a double-blind randomized trial showed that 3 mg/day MLT administered as a 3-month adjunctive therapy for mild-to-moderate ulcerative colitis was safe, potentially alleviating symptoms and improving quality of life [74]. A 12-week clinical trial demonstrated that a daily dose of 6 mg MLT improved anthropometric indices, liver enzymes, and inflammatory markers in patients with non-alcoholic fatty liver disease [75]. Another trial designed for patients with heart failure with reduced ejection fraction administered 10 mg/day MLT for 24 weeks to translate preclinical benefits while monitoring safety, and the results of this unique clinical trial might confirm MLT’s role as an adjunctive therapy not only for the primary disease, but also for the common comorbidities and complications of HF, along with patients’ overall health status [76]. MLT has also been exploratively applied in human renal disease models. It is found that 3 mg twice-daily MLT reduced acute kidney injury incidence in critically ill patients with vancomycin-induced nephrotoxicity [77]. Furthermore, a dose of 3 mg/day MLT (24 h pre-transplant to discharge) protected kidney transplant patients from ischemia–reperfusion injury [78]. In this study, we administered MLT at a dose of 10 mg/kg via oral gavage and observed its preventive effect on high-fructose-induced renal injury in mice. Although it is not feasible to directly convert this dose to humans, such preclinical research holds significant scientific value, as it provides essential mechanistic evidence supporting the potential renal protective role of MLT. Future studies may build upon the findings of this study to further explore a safe and effective dosage suitable for human application, thereby promoting the translation of melatonin from fundamental evidence to clinical practice.

## 5. Conclusions

**Figure 8:**
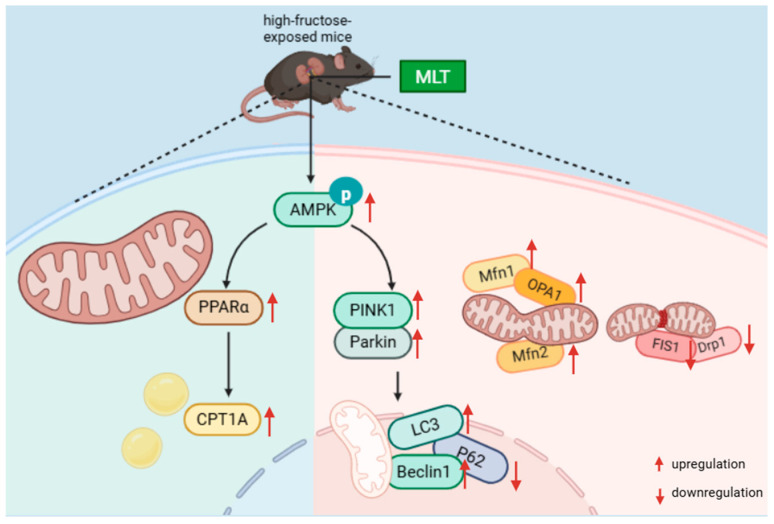
Potential alleviation mechanisms of MLT on high-fructose-induced renal injury in male mice.

MLT alleviates high-fructose-induced renal injury in male mice, which might be associated with the regulation of mitophagy and FAO (Figure 8). In the future, by integrating fundamental research with clinical evidence to optimize dosing strategies and systematically elucidate its renoprotective mechanisms, MLT holds promise as an important adjuvant in renal injury management.

## Data Availability

The datasets used and/or analyzed in the current study can be obtained from the corresponding author upon reasonable request. Data availability is restricted in accordance with laboratory policies and confidentiality agreements.

## Supplementary Materials

The following supporting information can be downloaded at https://www.mdpi.com/article/10.3390/nu18010068/s1, Table S1: Composition and energy of the study diets.

## Abbreviations

AMPK	AMP-activated protein kinase
ATP	adenosine triphosphate
BUN	urea nitrogen
CAT	catalase
CON	control
CPT1A	carnitine palmitoyltransferase 1A
Cre	creatinine
Drp1	dynamin-related protein 1
FAO	fatty acid oxidation
FIS1	fission mitochondrial
FRU	fructose
GSH-Px	glutathione peroxidase
H&E	Hematoxylin and Eosin
KIM-1	kidney injury molecule-1
LC3	Microtubule-associated protein light chain 3
MDA	malonaldehyde
MLT	melatonin
Mfn1	mitofusin1
Mfn2	mitofusin2
mtDNA	mitochondrial DNA
OPA1	optic atrophy 1
ORO	Oil Red O
PINK1	PTEN-induced kinase 1
PPARα	peroxisome proliferator-activated receptor-alpha
P62	Sequestosome-1
P-AMPK	phosphorylated AMP-activated protein kinase
ROS	reactive oxygen species
SOD	superoxide dismutase
TC	total cholesterol
TEM	transmission electron microscope
TG	triglyceride
TOM20	translocase of the outer membrane 20
UA	uric acid
