# Supplementary material for: Melatonin Alleviates High-Fructose-Induced Renal Injury in Male Mice, Which Might Be Associated with the Regulation of Mitophagy and Fatty Acid Oxidation
Source: Nutrients. 2025 Dec 25;18(1):68. doi: 10.3390/nu18010068 (PMC12787547; doi:10.3390/nu18010068)
Supplement: Supplementary file 1 [file nutrients-18-00068-s001.zip › nutrients-4032548-supplementary.pdf]

Supplementary Table S1. Composition of the study diets.

Diet component	CON	MLT	FRU	FRU+MLT
Casein (g/kg)	189.56	189.56	189.56	189.56
Corn starch (g/kg)	298.56	298.56	298.56	298.56
Maltodextrin (g/kg)	33.17	33.17	33.17	33.17
Sucrose (g/kg)	331.73	331.73	331.73	331.73
Cellulose (g/kg)	47.39	47.39	47.39	47.39
Lard (g/kg)	18.96	18.96	18.96	18.96
Soybean oil (g/kg)	23.70	23.70	23.70	23.70
L-cystine (g/kg)	2.84	2.84	2.84	2.84
Mineral mix (g/kg)	9.48	9.48	9.48	9.48
Vitamin Mix (g/kg)	9.48	9.48	9.48	9.48
Choline (g/kg)	1.90	1.90	1.90	1.90
Calcium carbonate(g/kg)	5.21	5.21	5.21	5.21
Calcium hydrogen phosphate (g/kg)	12.32	12.32	12.32	12.32
Potassium citrate (g/kg)	15.64	15.64	15.64	15.64
Folic acid (mg/kg)	2	2	2	2
